# Restless legs syndrome in chronic renal failure patients on dialysis

**DOI:** 10.4314/ahs.v23i3.88

**Published:** 2023-09

**Authors:** Carin Behrens van Tonder, Gina Joubert, Anand Moodley

**Affiliations:** 1 Department of Neurology, School of Clinical Medicine, Faculty of Health Sciences, University of the Free State, Bloemfontein, South Africa; 2 Department of Biostatistics, School of Biomedical Sciences, Faculty of Health Sciences, University of the Free State, Bloemfontein, South Africa; 3 Department of Neurology, School of Clinical Medicine, College of Health Sciences, University of KwaZulu-Natal, Durban, South Africa

**Keywords:** Restless legs syndrome, chronic renal failure, haemodialysis, peritoneal dialysis, symptoms

## Abstract

**Background:**

Restless legs syndrome (RLS) occurs in patients with chronic renal failure (CRF).

**Objectives:**

To determine the prevalence and morbidity of RLS in CRF patients on dialysis.

**Methods:**

This cross-sectional questionnaire-based study included 100 dialysis patients (50 on haemodialysis [HD]; 50 on peritoneal dialysis [PD]). A focused lower limb examination was done. Patients were classified with RLS when reporting uncomfortable feelings in their legs that improved with movement and worsened when resting and at night.

**Results:**

Gender distribution was equal. The median age was 43 (19–67) years. Six patients (HD and PD n=3 each) had international criteria-confirmed RLS. Twenty-four patients reported symptoms suggestive of RLS. Fourteen and 16 patients with RLS symptoms were on HD and PD, respectively. Sleep disturbances occurred in 43.3% (n=13) of patients with RLS symptoms, compared to 20.0% (n=20) of the large cohort. Sleep disturbances, peripheral sensory loss, chronic disease-related anaemia, increased urea and decreased albumin levels were more common among patients with RLS symptoms.

**Conclusion:**

RLS symptoms occurred in 30.0% of the entire cohort, although only 6.0% met the international criteria. The type of dialysis had no impact on the incidence of symptoms. Identifying RLS in patients with CRF on dialysis will allow for early intervention.

## Introduction

Restless legs syndrome (RLS) is characterised by abnormal sensations in the lower limbs and a persistent urge to move the limbs to relieve the feeling. It is more intense at night and while at rest.^1^,^2^ RLS affects the patients' sensorimotor system and results in disturbed sleep and negatively impacts their quality of life.^2^,^3^

The diagnosis is made when the patient's symptoms fulfil the International Restless Legs Syndrome Study Group (IRLSSG) criteria.^1^ The criteria include an uncomfortable feeling in the limbs that become more intense at rest and at night and improves on movement of the limbs. For a diagnosis of RLS, these symptoms should not be primarily due to another medical or behavioural condition.^1^,^4^ The uncomfortable symptoms described vary widely, but most patients experience creepy-crawly/shock-like sensations or itching.^5^

The prevalence of RLS differs markedly, which might be due to changes in diagnostic criteria over the years.^6^ Allen *et al.*^3^ reported a prevalence of 7.2%. In reviews of the literature, the prevalence ranged between 11% and 20%.^7^,^8^ Females are more affected than males, although an equal gender distribution has also been found.^3^,^9^,^10^ The African-American population was less likely to report RLS symptoms.^11^

No prevalence studies regarding RLS in South African populations or chronic renal failure (CRF) patients have been reported. A study done in Mozambique evaluating patients at a pain clinic revealed a 6.7% prevalence of RLS.^12^ No difference was found in the prevalence of RLS among urban and rural patients in a Tanzanian study and the prevalence in Asian and African patients appeared to be lower than in other population groups.^13^ None of these studies explicitly investigated patients with CRF and RLS.^12^,^13^ The South African context presents an additional challenge because of restricted resources, including dialysis, in public sector hospitals.

Restless legs syndrome is classified into primary (idiopathic) and secondary (symptomatic) types. However, this dichotomy has been justifiably challenged.^14^ The primary or idiopathic variety has a positive family history and genetic component in approximately 50% of cases.^5^,^9^ The inheritance pattern with RLS is autosomal dominant, presents earlier and includes more severe symptoms in subsequent generations.^15^,^16^ Environmental factors also contribute to the disorder. Genetic predisposition plays a major role in the prevalence of RLS in both the normal population and CRF patients.^17^

Secondary or symptomatic RLS more likely develops in older patients.^5^ Multiple chronic diseases are associated with secondary RLS and some of the most common include CRF, iron deficiency, diabetes mellitus and cardiovascular disease.^5^,^7^,^18^ Iron deficiency as a secondary cause of RLS has been well described. However, the pathophysiology is still unclear, despite several hypotheses having been postulated. The likelihood that brain iron metabolism may be affected, even when the serum iron and ferritin are normal, has been suggested.^19^ Other hypotheses include dopaminergic dysfunction, abnormal iron metabolism and central opiate systems dysfunction.^2^,^19^,^20^

Restless legs syndrome in CRF may be attributed to uraemia itself, iron deficiencies or treatment related to renal failure.^21^ In uraemic patients with RLS, the onset of the disease is considerably faster than in idiopathic RLS.^10^ Previous prevalence studies of RLS in CRF reported very large ranges (from 6.6 to 62%), but these studies were conducted before set criteria were in place.^2^,^9^

The study aimed to determine the prevalence of RLS in end-stage renal failure (ESRF) patients on dialysis at two Bloemfontein-based public sector hospitals in central South Africa, and its impact on their quality of life and emotional wellbeing.

## Methods

### Study design

A cross-sectional study was conducted on 100 dialysis patients.

### Setting and data collection

A physician-directed, questionnaire-based interview and physical examination were performed on patients who visited the Universitas Academic and Pelonomi Tertiary Hospitals Haemodialysis (HD) unit, as well as the Universitas Academic Hospital Peritoneal Dialysis (PD) unit. The questionnaire was compiled after reviewing literature for possible questions specific to the study population.^22^,^23^ Universitas Hospital is the only public sector hospital in the Free State Province with a PD unit. The HD units between the two hospitals serve approximately 70 patients per week, each three times per week. The PD unit manages 110–130 patients on a three-monthly basis if stable. Participants were recruited in the 10-month period ranging from 1 February 2020 to 30 November 2020.

We aimed to interview 50 HD and 50 PD chronic dialysis patients in end-stage kidney failure, and after these numbers were obtained no further recruitment took place. A consecutive sampling method was used to recruit patients. A pilot study was conducted on the first five patients interviewed to evaluate the feasibility of the study and the usability of the questionnaire. No changes to the questionnaire were required and the pilot study participants' data were included in the main analysis. All the questionnaires were translated into the three main local languages (English, Afrikaans and Sesotho) and a translator was used where needed.

The clinician-based interview and clinical examination findings were documented on an anonymised datasheet. The clinical examination aimed to exclude any signs mimicking symptoms of RLS. Clinical data were retrieved from the hospital database regarding the patients' medical history, comorbid diseases, current medication and blood test results. Dialysis patients undergo relevant blood tests on a regular basis, some monthly and others three-monthly as prescribed by the South African Renal Society.^24^

Patients were divided into three groups, namely patients with no RLS symptoms, patients with possible RLS symptoms (patients with symptoms suggestive of RLS but who did not qualify according to the IRLSSG criteria) and patients with RLS according to the IRLSSG criteria.^1^ Only the first four criteria were used for statistical purposes and subdivisions as the fifth criterion excluded the patient from recruitment into the study.

### Diagnosis

The IRLSSG criteria include the following characteristics of RLS and all the criteria must be met to make a definitive diagnosis of RLS:^1^

- an urge to move the legs is usually (but not always) accompanied by uncomfortable and unpleasant sensations in the legs;
- the urge to move the legs and the accompanying unpleasant sensations start or get worse during rest or when inactive, such as lying down or sitting;
- the urge to move the legs and any accompanying unpleasant sensations are partially or totally relieved by movement, such as walking or stretching, as long as the activity continues;
- the urge to move the legs and accompanying unpleasant sensations during rest or inactivity only occur or become worse in the evening/at night than during the day; and
- these experiences are not primarily related to symptoms of another medical condition or behaviour (e.g., myalgia, venous stasis, leg oedema, arthritis, leg cramps, positional discomfort, habitual foot tapping).

As part of the questionnaire, we enquired whether either the symptoms of RLS or CRF had disturbed their sleep. Sleep disturbances were identified if the patient complained that the symptoms of either condition prevented them from falling asleep, or disturbed them while sleeping. The patients were asked if the symptoms in their legs made them feel worried or depressed, and whether these feelings and other mood changes were mostly attributed to their leg symptoms or renal failure diagnosis.

### Statistical analysis

Results were summarised by frequencies and percentages (categorical variables) and medians and percentiles (numerical variables due to skew distributions). Subgroups were compared using chi-squared or Fisher's exact tests for categorical variables and Mann-Whitney tests for numerical variables. A p value of <0.05 indicated statistical significance. Analyses were performed by the Department of Biostatistics, University of the Free State, using statistical analysis software SAS, Version 9.4 (SAS Institute Inc.; Cary, NC).

### Ethical considerations

Ethical approval to conduct the study was obtained from the University of Free State Health Sciences Research Ethics Committee (ref. no. UFS-HSD2019/2025/2403). All data were anonymised to ensure confidentiality after obtaining informed consent from the patients.

## Results

Fifty-two patients in this study were female. The median age was 43 years (range 19–63 years). The median duration on dialysis was five years (range 1–34 years). The racial distribution of the group is presented in Table 1. Based on IRLSSG criteria, six patients were diagnosed with RLS (95% CI 2.2%; 12.6%) as they answered all four questions affirmatively. Some of the answers of a number of the other patients suggested that they might also have RLS symptoms, but did not strictly fulfil the criteria. Fourteen patients had three criteria and ten patients had either one or two criteria suggestive of RLS. The remaining 70 patients had no symptoms indicative of RLS. All the groups had an equal gender and mode of dialysis distribution (Table 1).

**Table 1:** Demographic information of chronic renal failure (CRF) patients with and without symptoms of restless legs syndrome (RLS)

Variable	No RLS symptoms (n = 70)	Total with RLS symptoms (n = 30)	Patients with symptoms suggestive of RLS (n = 30)			Total group of CRF patients (n = 100)	p-value
			One or two symptoms (n = 10)	Three symptoms (n = 14)	Criteria-defined RLS (n = 6)		
	n (%)	n (%)	n (%)	n (%)	n (%)	n (%)	
**Race**							
**Black**	65 (92.9)	27 (90.0)	9 (90.0)	14 (100)	4 (66.7)	92 (92.0)	0.003*
**White**	0 (0)	1 (3.3)	0 (0)	0 (0)	1 (16.7)	1 (1.0)	
**Mixed ethnicity**	5 (7.1)	2 (6.7)	1 (10.0)	0 (0)	1 (16.7)	7 (7.0)	
**Gender**							
**Male**	33 (47.1)	15(50.0)	6 (60.0)	6 (42.9)	3 (50.0)	48 (48.0)	
**Female**	37 (52.9)	15 (50.0)	4 (40.0)	8 (57.1)	3 (50.0)	52 (52.0)	
**Mode of dialysis**							
**Haemodialysis (HD)**	36 (51.4)	14 (46.7)	8 (80.0)	3 (21.4)	3 (50.0)	50 (50.0)	
**Peritoneal dialysis (PD)**	34 (48.6)	16 (53.3)	2 (20.0)	11 (78.6)	3 (50.0)	50 (50.0)	
**Years living with a diagnosis of CRF**							
**Median**	4.0	–	9.5	5.5	8.5	5.5	0.081#; 0.119*
**IQR**	2.0 – 9.0	–	3.0 – 18.0	1.0 – 10.0	6.0 – 13.0	2.0 - 10.0	
**Years on dialysis**							
**Median**	4.0	–	9.5	5.5	8.0	5.0	0.046#; 0.099*
**IQR**	2.0 – 8.0	–	3.0 – 18.0	1.0 – 10.0	6.0 – 13.0	2.0 – 10.0	

IQR: interquartile range

* p-value for differences between patients with criteria-defined RLS (n = 6) and patients without symptoms (n = 70)

# p-value for differences between all patients with symptoms suggestive of RLS (n = 30) and patients without symptoms (n = 70).

Patients with criteria-defined RLS had a median age of 45 years (range 29–49 years) with a median of 8 years on dialysis (range 6–13 years). The median years of dialysis in the group with symptoms (7.5 years) compared to the group without any symptoms (4 years) differed significantly (p=0.046).

**Figure 1:**
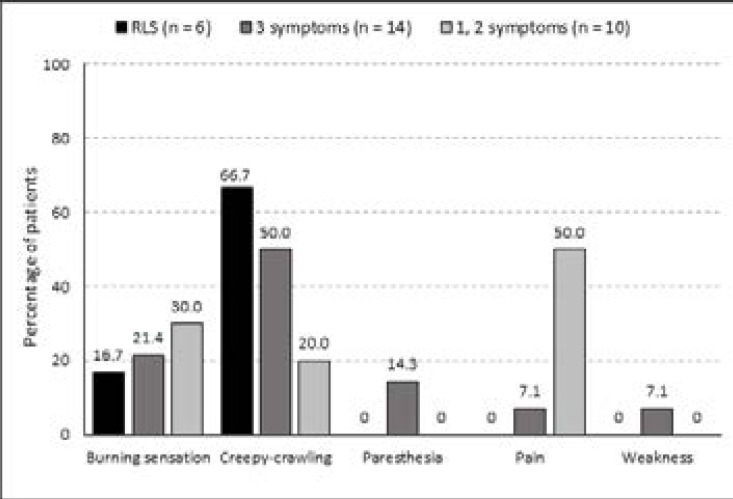
Symptoms reported by chronic renal failure CRF) patients who experienced an uncomfortable feeling in their legs (n=30)

The patients with suggestive RLS symptoms offered wide descriptions of their symptoms (Figure 1). The patients with criteria-defined RLS described it mainly as a burning or a creepy-crawling sensation. Other symptoms included paresthesia, pain or weakness. Various leg symptoms were reported (Table 2). The prevalence of pain was significantly higher in patients with criteria-defined RLS and symptomatic patients compared to asymptomatic patients.

**Table 2:** Leg symptoms and cause of depressive symptoms among chronic renal failure (CRF) patients with and without symptoms of restless legs syndrome (RLS)

Variable	No RLS symptoms (n=70)	Total with symptoms (n=30)	Patients with symptoms suggestive of RLS (n=30)			Total group of CRF patients (n=100)	p-value
			One or two symptoms (n=10)	Three symptoms (n=14)	Criteria-defined RLS(n=6)		
	n (%)	n (%)	n (%)	n (%)	n (%)	n (%)	
**Leg symptoms**							
**Pain**	14 (20.0)	18 (60.0)	8 (80.0)	5 (35.7)	5 (83.3)	32.0 (32.0)	0.003*; < 0.001^#^
**Joint pains**	18 (25.7)	13 (43.3)	5 (50.0)	6 (42.9)	2 (33.3)	31 (31.0)	0.651*; 0.100^#^
**Cramps**	33 (47.1)	16 (53.3)	7 (70.0)	7 (50.0)	2 (33.3)	49 (49.0)	0.681*; 0.614^#^
**Weakness**	18 (25.7)	14 (46.7)	4 (40.0)	6 (42.9)	4 (66.7)	32 (32.0)	0.055*; 0.060^#^
**Cause of depressive symptoms**							
**Rena failure**	23 (32.9)	14 (46.7)	5 (50.0)	6 (42.9)	3 (50.0)	37 (37.0)	0.027*; < 0.001^#^
**Leg symptoms**	0 (0)	9 (30.0)	4 (40.0)	4 (28.6)	1 (16.7)	9 (9.0)	
**No symptoms of depression**	47 (67.1)	7 (23.3)	1 (10.0)	4 (28.6)	2 (33.3)	54 (54.0)	

IQR: interquartile range

* p-value for differences between patients with criteria-defined RLS (n = 6) and patients without symptoms (n = 70)

# p-value for differences between all patients with symptoms suggestive of RLS (n = 30) and patients without symptoms (n = 70).

**Figure 2:**
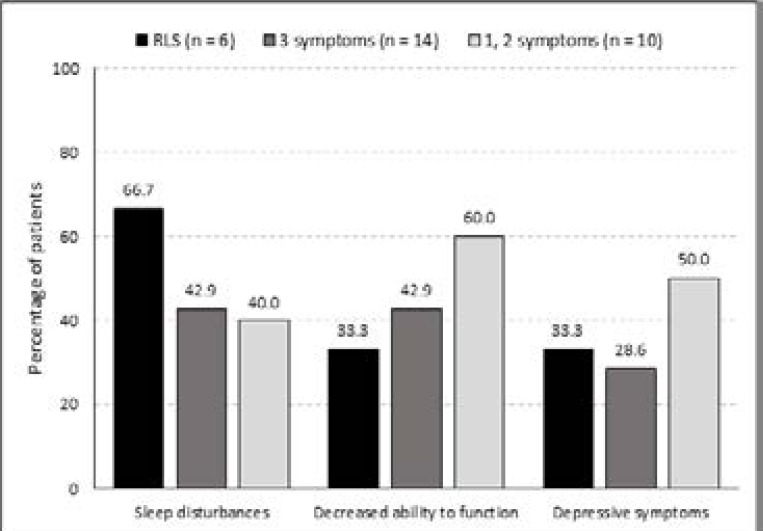
Sleep disturbances, decreased ability to function and depressive symptoms reported by chronic renal failure (CRF) patients who experienced symptoms of restless legs syndrome (RLS)

Sleep disturbances occurred in 20% of all the patients. However, 43% of the patients with RLS symptoms mentioned sleep problems (p=0.067). Two-thirds (66%) of patients with criteria-defined RLS had sleep disturbances due to RLS, compared to the suspected cases of whom 41% reported sleep disturbances. In the criteria-defined RLS patients, 33% had a decreased ability to perform their duties, compared to 50% (12/24) in the suspected RLS patients. Patients with RLS symptoms mentioned feeling stressed or depressed (Figure 2). In patients with suspected RLS 37.5% (9/24) mentioned feeling stressed or depressed about their leg symptoms, which was similar in the confirmed RLS patients with 33% (2/6).

In patients with suggestive RLS symptoms, 76.7% (23/30) had a depressed mood, of whom 60.9% (14/23) primarily ascribed it to CRF, while 39.1% (9/23) indicated that it was due to their RLS symptoms (Table 2).

On clinical examination, 14 (14.0%) of the 100 patients had peripheral sensory deficits (nine had pain and temperature loss and five had proprioceptive loss). In the six patients with criteria-defined RLS, only one had decreased pain sensation (Table 3). Leg oedema was present in 27 (27.0%) of all the CRF patients and in two (33.3%) patients with criteria-defined RLS (n=6). No statistically significant differences were found between the patients with criteria-defined RLS and those without symptoms, and between patients with symptoms and those without symptoms.

**Table 3:** Clinical leg examination of chronic renal failure (CRF) patients with and without symptoms of restless legs syndrome (RLS)

Clinical feature	No RLS symptoms (n=70)		Total with RLS symptoms (n=30)		Patients with symptoms suggestive of RLS (n=30)						Total group of CRF patients (n=100)	
					One or two symptoms (n=10)		Three symptoms (n=14)		Criteria-defined RLS (n=6)			
	n (%)		n (%)		n (%)		n (%)		n (%)		n (%)	
	RL	LL	RL	LL	RL	LL	RL	LL	RL	LL	RL	LL
**Atrophy**	0 (0)	0 (0)	0 (0)	0 (0)	0 (0)	0 (0)	0 (0)	0 0 (0)	0 (0)	0 (0)	0 (0)	0 (0)
**Discolouration**	2 (2.6)	2 (2.6)	4 (13.3)	2 (6.7)	2 (20.0)	1 (10.0)	2 (14.3)	1 (7.1)	0 (0)	0 (0)	6 (6.0)	4 (4.0)
**Oedema**	16 (22.8)	6 (22.8)	11 (36.7)	10 (33.3)	3 (30.0)	2 (20.0)	6 (42.9)	6 (42.9)	2 (33.3)	2 (33.3)	27 (27.0)	26 (26.0)
**Knee flexion (some weakness 4/5)**	3 (4.3)	3 (4.3)	3 (10.0)	3 (10.0)	0 (0)	0 (0)	2 (14.3)	2 (14.3)	1 (16.7)	1 (16.7)	6 (6.0)	6 (6.0)
**Knee extension (some weakness 4/5)**	3 (4.3)	3 (4.3)	3 (10.0)	3 (10.0)	0 (0)	0 (0)	2 (14.3)	2 (14.3)	1 (16.7)	1 (16.7)	6 (6.0)	6 (6.0)
**Plantar flexion (some weakness 4/5)**	1 (1.4)	1 (1.4)	1 (3.3)	2 (6.7)	0 (0)	1 (10.0)	1 (7.1)	1 (7.1)	0 (0)	0 (0)	2 (2.0)	3.(3.0)
**Dorsi-flexion (some weakness 4/5)**	1 (1.4)	1 (1.4)	1(3.3)	1 (3.3)	0 (0)	0 (0)	1 (7.1)	1 (7.1)	0 (0)	0 (0)	2 (2.0)	2.(2.0)
**Achilles tendon reflex abnormal**	36 (51.4)	37 (52.9)	13 (43.3)	13 (43.3)	4 (40.0)	4(40.0)	9 (64.3)	9 (64.3)	2 (33.3)	2 (33.3)	51 (51.0)	52 (52.0)
**Pinprick abnormal**	6 (8.6)	6 (8.6)	4 (13.3)	5 (16.7)	1 (10.0)	1 (10.0)	2 (14.3)	3 (21.4)	1 (16.7)	1 (16.7)	10 (10.0)	11 (11.0)
**Soft touch abnormal**	2 (2.6)	2 (2.6)	1 (3.3)	1 (3.3)	0 (0)	0 (0)	1 (7.1)	1 (7.1)	(0)	(0)	3 (3.0)	3 (3.0)
**Vibration sense abnormal**	3 (4.3)	3 (4.3)	1 (3.3)	2 (6.7)	0 (0)	0 (0)	1 (7.1)	2 (14.3)	0 (0)	0 (0)	4 (4.0)	5 (5.0)
**Temperature abnormal**	5 (7.1)	5 (7.1)	4 (13.3)	4 (13.3)	1 (10.0)	1 (10.0)	3 (21.4)	3 (21.4)	0 (0)	0 (0)	9 (9.0)	9 (9.0)
**Joint position sense abnormal**	3 (4.3)	3 (4.3)	1 (3.3)	1 (3.3)	0 (0)	0 (0)	1 (7.1)	1 (7.1)	0 (0)	0 (0)	4 (4.0)	4 (4.0)
**Glove-stocking sensory loss**	5 (7.1)	5 (7.1)	4 (13.3)	4 (13.3)	1 (10.0)	1 (10.0)	2 (14.3)	2 (14.3)	1 (16.7)	1 (16.7)	9 (9.0)	9 (9.0)
**Monofilament test abnormal**	5 (7.1)	5 (7.1)	3 (10.0)	4 (13.3)	0 (0)	1 (10.0	3 (21.4)	3 (21.4)	0 (0)	0 (0)	8 (8.0)	9 (9.0)

RL: right leg; LL: left leg

The blood test results (Table 4) showed differences between the three groups. The median haemoglobin (Hb) level of the group with no RLS symptoms was 11.0 g/dL (interquartile range [IQR] 9.9–12.1 g/dL), compared to those with suggestive RLS symptoms who had a median Hb level of 9.6 g/dL (IQR 8.0–11.0 g/dL; p=0.008). Comparing patients without symptoms to those with criteria-defined RLS symptoms, the difference was not significant, with a median Hb level of 10.7 g/dL (IQR 8.4–12.5) in the latter group (p=0.743).

**Table 4:** Comparison of blood test results of chronic renal failure (CRF) patients with and without symptoms of restless legs syndrome (RLS)

Variable	No symptoms (n=70)	Total with symptoms (n=30)	Patients with symptoms suggestive of RLS (n=30)			Total group of CRF patients (n=100)	p-value
			One or two symptoms (n=10)	Three symptoms (n=14)	Criteria-defined RLS (n=6)		
**Haemoglobin (g/dL)**							
**Median**	11.0	9.6	8.95	9.7	10.7	10.5	0.008*; 0.743^#^
**IQR**	9.9–12.1	8.0–11.0	7.7–10.2	8.5–10.4	8.4-12.5	9.05-12.0	
**Hematocrit (L/L)**							
**Median**	0.351	0.290	0.297	0.298	0.332	0.337	0.024*; 0.985^#^
**IQR**	0.304–0.380	0.259–0.347	0.23–0.343	0.267–0.327	0.280-0.410	0.281-0.378	
**Iron (µmol/L)**							
**Median**	11.0	11.0	12.0	11.5	8.75	11.0	0.743*; 0.142^#^
**IQR**	9.0–15.0	8.0–15.0	7.0–15.7	9.0–16.0	5.9-11.0	8.8-15.0	
**Transferrin (g/L)**							
**Median**	1.84	1.78	1.90	1.72	1.75	1.83	0.236*; 0.307^#^
**IQR**	1.65–2.17	1.64–2.03	1.75–2.38	1.58–2.00	1.60-1.86	1.64-2.12	
**Transferrin saturation (%)**							
**Median**	23.0	23.0	23.0	25.5	19.5	23.0	0.761*; 0.307^#^
**IQR**	17.0–29.0	18.0–30.0	20.0–27.0	18.0–36.0	13.0-23.0	17.0-30.0	
**Calcium (mmol/L)**							
**Median**	2.30	2.33	2.65	2.31	2.51	2.30	0.813*; 0.656^#^
**IQR**	2.13–2.47	2.10–2.50	2.02–2.45	2.10–2.45	2.34-2.58	2.16-2.48	
**Phosphate (mmol/L)**							
**Median**	1.65	1.41	1.44	1.35	1.41	1.21	0.107*; 0.686^#^
**IQR**	0.82–1.57	1.05–2.19	0.76–2.19	1.07–2.2	0.78-1.51	0.84-1.61	
**Albumin (g/L)**							
**Median**	31.0	29.0	31.5	25.0	30.5	30.0	0.047*; 0.862^#^
**IQR**	27.0–35.0	25.0–33.0	26.0–35.0	20.0–30.0	29.0-34.0	25.5-35.0	
**Alkaline phosphatase (ALP) (U/L)**							
**Median**	130.5	118.0	112.5	125.5	111.5	121.5	0.562*; 1.00^#^
**IQR**	88.0–186.0	82.0–230.0	92.0–140.0	72.0–230.0	84.0–262.0	85.5-192.0	
**Urea (µmol/L)**							
**Median**	13.8	20.9	18.4	23.1	16.4	15.8	< 0.001*; 0.563^#^
**IQR**	6.2–18.9	15.9–24.8	8.8–22.7	19.6–30.0	6.2–22.2	8.35-21.3	
**Creatinine (µmol/L)**							
**Median**	845.5	1127.0	937.5	1188.0	1050.0	908.0	0.014*; 0.232^#^
**IQR**	676.0–1123.0	908.0–1316.0	567.0–1256.0	1120.0–1469.0	1036.0–1093.0	700.5-1238.5	

IQR: interquartile range.

* *p*-value for differences between patients with criteria-defined RLS (*n*=6) and patients without symptoms (*n*=70)

# *p*-value for differences between all patients with symptoms suggestive of RLS (*n*=30) and patients without symptoms (*n*=70).

The median iron level in the group without symptoms was 11.0 umol/L (IQR 9.0–15.0), compared to those with criteria-defined RLS (median iron level 8.75 µmol/L; IQR 5.9–11.0; p=0.142). The median transferrin saturation in the group without symptoms was 23% (IQR 17–29), compared to those with definite symptoms (median 19.5%; iQr 13–23; p=0.307) (Table 4).

Patients with criteria-defined RLS had a median urea value of 16.35 umol/L (IQR 6.2–18.9), compared with the patients with no symptoms who had median value of 13.8 umol/L (IQR 6.2–21.3; p=0.563). When comparing the groups with suggestive RLS symptoms to those without symptoms, the median value for those with symptoms was 20.9 umol/L (IQR15.9–24.8; p<0.001) (Table 4).

The group without symptoms had a median albumin value of 31 g/L (IQR 27.0–35.0), compared to patients with criteria-defined RLS, with a median value of 30.5 g/L (IQR 29.0–34.0; p=0.862). Patients with symptoms suggestive of RLS had a median albumin value of 29.0 g/L (IQR 25.0–33.0; p=0.047).

Renal dialysis patients had several other medical conditions (Table 5), the most common being hypertension, constipation, hyperparathyroidism, HIV, gout and cardiac disease. A comparison between the asymptomatic group and all the patients with suggestive RLS symptoms, and between the asymptomatic group and those with criteria-define RLS, showed no significant difference regarding comorbidities.

**Table 5:** Comorbid conditions among chronic renal failure (CRF) patients with and without symptoms of restless legs syndrome (RLS)

Variable	No RLS symptoms (n=70)	Total with RLS symptoms (n=30)	Patients with symptoms suggestive of RLS (n = 30)			Total group of CRF patients (n=100)
			One or two symptoms (n=10)	Three symptoms (n=14)	Criteria-defined RLS (n=6)	
	n (%)	n (%)	n (%)	n (%)	n (%	n (%)
**Hypertension**	69 (98.6)	30 (100)	10 (100)	14 (100)	6 (100)	99 (99.0)
**Constipation**	27 (38.6)	15 (50.0)	4 (40.0)	8 (57.1)	3 (50.0)	42 (42.0)
**Hyperparathyroidism**	27 (38.6)	10 (33.3)	3 (30.0)	4 (28.6)	3 (50.0)	37 (37.0)
**HIV**	21 (30.0)	7 (23.3)	2 (30.0)	4 (28.6)	0 (0)	28 (28.0)
**Gout**	15 (21.4)	8 (26.7)	0 (0)	5 (35.7)	3 (50.0)	23 (23.0)
**Cardiac disease**	10 (14.3)	4 (13.3)	2 (20.0)	2 (14.3)	0 (0)	14 (14.0)
**Superior vena cava syndrome**	6 (8.6)	1 (3.3)	0 (0)	1 (7.1)	0 (0)	7 (7.0)
**Hypothyroidism**	3 (4.3)	2 (6.7)	0 (0)	1 (7.1)	1 (16.7)	5 (5.0)
**Diabetes**	2 (2.9)	1 (3.3)	0 (0)	1 (7.1)	0 (0)	3 (3.0)
**Encapsulating peritoneal sclerosis**	2 (2.9)	1 (3.3)	0 (0)	1 (7.1)	0 (0)	3 (3.0)
**Previous Kidney transplant**	1 (1.4)	2 (6.7)	0 (0)	1 (7.1)	1 (16.7)	3 (2.0)
**Epilepsy**	1 (1.4)	1 (3.3)	1 (10.0)	0 (0)	0 (0)	2 (2.0)
**Pulmonary hypertension**	1 (1.4)	1 (3.3)	0 (0)	1 (7.1)	0 (0)	2 (2.0)
**Major depression**	2 (2.9)	(0)	0 (0)	0 (0)	0 (0)	2 (2.0)
**Systemic lupus erythematosus**	1 (1.4)	(0)	0 (0)	0 (0)	0 (0)	1 (1.0)
**Hyperthyroidism**	1 (1.4)	(0)	0 (0)	0 (0)	0 (0)	1 (1.0)
**Previous pulmonary emboli**	1 (1.4)	(0)	0 (0)	0 (0)	0 (0)	1 (1.0)
**Liver disease**	0 (0)	7 (23.3)	0 (0)	1 (7.1)	0 (0)	1 (1.0)
**ANCA vasculitis**	1 (1.4)	(0)	0 (0)	0 (0)	0 (0)	1 (1.0)
**Polycystic kidney disease**	0 (0)	1 (0.3)	1 (10.0)	0 (0)	0 (0)	1 (1.0)
**Renal cell carcinoma**	1 (1.4)	(0)	0 (0)	0 (0)	0 (0)	1 (1.0)
**FSGS**	0 (0)	1 (3.3)	0 (0)	0 (0)	1 (16.7)	1 (1.0)
**Hepatitis C**	1 (1.4)	(0)	0 (0)	0 (0)	0 (0)	1 (1.0)
**Hepatitis B**	0 (0)	1 (3.3)	0 (0)	1 (7.1)	0 (0)	1 (1.0)
**Deep vein thrombosis**	1 (1.4)	(0)	0 (0)	0 (0)	0 (0)	1 (1.0)

ANCA: antineurtophil cytoplasm antibodies; FSGS: focal segmental glomerulonephritis; HIV: human immunodeficiency virus.

Patients were on multiple pharmacological agents to treat their comorbidities (Table 6). Ninety-seven (97.0%) of the total group were on antihypertensive treatment. Other medications included erythropoietin (87.0% of the total group; 100% of the criteria-defined RLS group), phosphate binders (76.0% of the total group; 83.3% of the criteria-defined RLS group), iron supplements (74.0% of the total group; 66.7% of the criteria-defined RLS group), calcium supplements (69.0% of the total group; 100% of the criteria-defined RLS group) and proton pump inhibitors (PPI) (65.0% of the total group; 100% of the criteria-defined RLS group).

**Table 6:** Medications used by chronic renal failure (CRF) patients with and without symptoms of restless legs syndrome (RLS)

Medication	No RLS symptoms (n=70)	Total with RLS symptoms (n = 30)	Patients with symptoms suggestive of RLS (n=30)			Total group of CRF patients (n = 100)
			One or two symptoms (n=10)	Three symptoms (n=14)	Criteria-defined RLS (n=6)	
	n (%)	n (%)	n (%)	n (%)	n (%)	n (%)
**Antihypertensive agent**	68 (97.2)	29 (96.7)	10 (100)	13 (92.6)	6 (100)	97 (97.0)
**Erythropoietin**	50 (85.7)	27 (90.3)	10 (100)	11 (78.6)	6 (100)	87 (87.0)
**Phosphate binders**	51 (72.9)	25 (83.3)	8 (80.0)	12 (85.7)	5 (83.3)	76 (76.0)
**Iron supplements**	51 (72.9)	23 (76.7)	7 (70.0)	12 (85.7)	4 (66.7)	74 (74.0)
**Calcium supplements**	45 (64.3)	24 (80.0)	7 (70.0)	11 (78.6)	6 (100)	69 (69.0)
**Proton pump inhibitors**	45 (64.3)	20 (66.7)	5 (50.0)	9 (64.3)	6 (100)	65 (65.0)
**Laxatives**	26 (37.1)	15 (50.0)	4 (40.0)	8 (57.1)	3 (50.0)	41 (41.0)
**Pain medications**	25 (35.7)	11 (36.7)	3 (30.0)	7 (50.0)	1 (16.7)	36 (36.0)
**Statin**	24 (34.3)	9 (30.0)	2 (20.0)	3 (21.4)	4 (66.7)	33 (33.0)
**Antiretroviral treatment**	21 (30.0)	8 (26.7)	3 (30.0)	5 (35.7)	0 (0)	29 (29.0)
**Alopurinol**	15 (21.4)	8 (26.7)	0 (0)	5 (35.7)	3 (50)	23 (23.0)
**Antiplatelet therapy**	10 (14.3)	5 (16.7)	3 (30.0)	1 (7.1)	1 (16.7)	15 (15.0)
**Carvedilol**	8 (11.4)	2 (6.7)	0 (0)	2 (14.3)	0 (0)	10 (10.0)
**Antidepressive therapy**	5 (7.1)	6 (20.0)	3 (30.0)	2 (14.3)	1 (16.7)	11 (11.0)
**Warfarin**	4 (5.7)	2 (6.7)	1 (10.0)	1 (7.1)	0 (0)	6 (6.0)
**Corticosteroids**	2 (2.9)	2 (6.7)	1 (10.0)	1 (7.1)	0 (0)	4 (4.0)
**Levothyroxine**	3 (4.3)	1 (3.3)	0 (0)	1 (7.1)	0 (0)	4 (4.0)
**Antidiabetic treatment**	2 (2.9)	1 (3.3)	0 (0)	1 (7.1)	0 (0)	3 (3.0)
**Sedative treatment**	3 (4.3)	0.0 (0)	0 (0)	0 (0)	0 (0)	3 (3.0)
**Benzodiazepines**	1 (1.4)	2 (6.7)	0 (0)	1 (7.1)	1 (16.7)	3 (3.0)
**Anti-epileptic treatment**	1 (1.4)	1 (3.3)	1 (10.0)	0 (0)	0 (0)	2 (2.0)
**Colchicine**	0 (0)	2 (6.7)	0 (0)	1 (7.1)	1 (16.7)	2 (2.0)
**Anti-osteoporosis treatment**	2 (2.9)	0 (0)	0 (0)	0 (0)	0 (0)	2 (2.0)
**Pyridoxine**	1 (1.4)	1 (3.3)	0 (0)	7.1 (1)	0 (0)	2 (2.0)
**Mycofenitol mofilate**	1 (1.4)	0 (0)	0 (0)	0 (0)	0 (0)	1 (1.0)

Comparing the group of criteria-defined RLS patients with those without symptoms, the most notable difference was in patients with RLS taking colchicine (p=0.088) and a combination of azathioprine with cyclosporine (p=0.078). More patients with suggestive RLS symptoms used colchicine than the asymptomatic group, although the difference was not statistically significant (p=0.078). More patients with suggestive RLS symptoms were on antidepressants than the asymptomatic group, although the difference between the groups was not significant (p=0.082).

## Discussion

The study confirmed a 6.0% prevalence of criteria-defined RLS in CRF patients on dialysis, but a total of 30.0% of the patients experienced some symptoms described in the IRLSSG criteria.^1^ This prevalence was lower than previous studies reporting a criteria-defined RLS prevalence of 7.5% to 24%.^6^,^25^–28 The reason for the lower rate might be due to studies done before the IRLSSG criteria were accepted, or the financially rationalised selection of dialysis patients in South African state sector.

Our patients were equally distributed regarding gender, which was similar to findings of Aritake-Okada *et al.*,^29^ although Brzuszek *et al.*^28^ reported a higher female prevalence. We also found an equal distribution of patients with and without RLS symptoms pertaining to dialysis modality, which differed from the findings of De Menezes *et al.* who reported a slightly higher prevalence of RLS in PD patients (24.7% versus 19.3% of patients on HD).^27^

Patients with suggestive RLS symptoms had been on dialysis longer than those without symptoms, with a statistically significant difference between the groups. Rohani *et al.* reported similar results in a study on HD patients.^30^ This finding might be due to the longer duration of uraemia, or longer periods of anaemia of chronic disease or iron deficiency.

Peripheral neuropathy and oedema are very common features of CRF.^31^–^33^ Oedema is commonly due to inter-dialytic weight gain and chronic fluid overload. Therefore, finding 27.0% of patients with pedal oedema in the total group of 100 CRF patients was not surprising. Clinical peripheral neuropathy was found in 14.0% of the CRF patients in this study, which was less than the occurrence of peripheral neuropathy in 50–60% of patients reported previously.^34^ Peripheral neuropathies in CRF may have multiple aetiologies, although further evaluation and tests are needed for their exact identification.^33^ However, further assessment using nerve conduction studies, which probably would have revealed more subclinical neuropathies, was beyond the scope of this study.

Sleep disturbances, especially insomnia and related depressive symptoms, were more common in the group with RLS symptoms. The prevalence of sleep disturbances among our patients was similar to that reported by Almeida Araujo *et al.*^35^ (69.8%) and Gade *et al.*^36^ (71%). Sleep disturbances, such as insomnia, obstructive sleep apnoea and hypersomnolence, were common in CRF patients, but insomnia in particular was aggravated when combined with RLS symptoms. Matar *et al.* reported that sleep deprivation does contribute to aggravated RLS symptoms.^33^

Szentkiralyi *et al.* reported depressive mood changes in 56% of CRF patients with RLS,^37^ compared to our results of 43–50%. Patients with CRF have to deal with having a serious chronic disease, but also having RLS may play a contributory role to depressive mood disturbances. Patients with suggestive RLS symptoms were more frequently using antidepressive medication, which concurred with previous findings.^38^ Patients with CRF on dialysis often suffer from depression and therefore receive long-term antidepressant treatment regardless of the co-occurrence of RLS.

The most significant differences found between the groups with suggestive RLS symptoms and those without any symptoms were in the haemoglobin, albumin, urea and creatinine levels. The group with RLS symptoms had lower haemoglobin and albumin levels, but higher urea and creatinine levels.

Uraemic RLS has been well described.^21^ We observed a significant difference between the group with suggestive RLS symptoms and patients without RLS symptoms, where the symptomatic group had higher urea levels (p<0.001). Lower haemoglobin levels are more prevalent in RLS,^2^ which was also the case in this study among patients with RLS symptoms (p=0.008). The albumin levels in the patients with suggestive RLS symptoms were slightly lower than in those without symptoms (p=0.047). Olgun Yazar *et al.* concluded that lower albumin was not only related to RLS symptoms, but also to its severity.^39^ In a recent study by Brzuszek *et al.*, however, no correlation with hypoalbuminaemia could be confirmed.^28^ This might be due to chronic inflammation, poor absorption or chronic anorexia associated with renal failure. Exactly how lower albumin levels contribute to RLS has not been well defined and is an area for future research, as an association was noted nonetheless.

Patients with suggestive RLS symptoms were on antihypertensive treatment, laxatives and general renal bone disease treatment, which was to be expected, but a large group of the patients also received treatment for gout. Both groups were on allopurinol, but only patients with suggestive RLS symptoms were on colchicine (p=0.078). However, it was only 2/6 patients and further research is necessary. A clinical trial on patients taking colchicine who develop RLS is currently in progress, with preliminary results showing that 53.3% of patients > 60 years of age taking colchicine have RLS.^40^

None of the patients in our study were on treatment for RLS, although all the symptomatic patients would benefit from management, whether medical or interventional. For patients on HD, Kerr *et al.*^41^ proposed cooler dialysate at 36.5°C instead of 37°C, although the symptoms only improved after a few months of using cooler dialysate.^41^

## Limitations

This study has a number of limitations. Only 100 patients were included and a more extensive study population might yield different results. A validated questionnaire in a dialysis population might improve results. Patients with peripheral neuropathy were advised to attend the neurology clinic, but their neurological findings were not considered in the analysis of the results, as it was beyond the scope of the study. Nerve conduction studies were not done to determine the presence of subclinical neuropathies. Only patients on dialysis in the public sector were recruited. Patients with chronic renal disease (CRD) who did not qualify for dialysis based on the treatment policy of the health department were not recruited for practical reasons. The International RLS rating scale could have been used to evaluate the RLS severity. A recent study^28^ compared neutrophil count to the prevalence of RLS, which could easily have been included in this study. However, the current study was conducted before the publication of these findings in 2022.^28^

## Conclusion and recommendations

The prevalence of criteria-defined RLS in the CRF study population was 6.0%, but 30.0% of the patients' experienced symptoms of RLS. Thirty-three percent of the patients reported an impact on daily activities and 76% reported depressive symptoms, compared to 33% of those without RLS symptoms. Anaemia associated with chronic disease, higher urea levels and lower albumin levels were more common in patients with symptoms of RLS. Longer duration of dialysis and a longer history of CRF were associated with more symptoms of RLS. There were no statistically significant associations between the mode of dialysis, gender or race and RLS symptoms.

We recommend regular screening of CRF patients for RLS symptoms. Symptomatic patients should be evaluated for comorbidities, appropriately managed and considered for medical treatment due to the psychosocial impact of RLS. The prevalence of RLS is notably lower when the IRLSSG criteria are used to diagnose RLS, but this study showed that even one or two symptoms of RLS are sufficient to cause impairment and therefore probably warrants appropriate treatment.

## Data Availability

All data generated and analysed during this study are presented in the article. Further enquiries can be directed to the corresponding author.

## References

[1] International Restless Legs Syndrome Study Group (IRLSSG) (2012). Diagnostic criteria.

[2] Kavanagh D, Siddiqui S, Geddes CC (2004). Restless legs syndrome in patients on dialysis. American Journal of Kidney Diseases.

[3] Allen RP, Walters AS, Montplaisir J (2005). Restless legs syndrome prevalence and impact: REST general population study. Archives of Internal Medicine.

[4] Walters AS, Frauscher B, Allen R (2014). Review of quality-of-life instruments for the restless legs syndrome/Willis-Ekbom disease (RLS/WED): critique and recommendations. Journal of Clinical Sleep Medicine.

[5] Cotter PE, O'Keeffe ST (2006). Restless leg syndrome: is it a real problem?. Therapeutics and Clinical Risk Management.

[6] Lin Z, Zhao C, Luo Q, Xia X, Yu X, Huang F (2016). Prevalence of restless legs syndrome in chronic kidney disease: a systematic review and meta-analysis of observational studies. Renal Failure.

[7] Innes K, Selfe TK, Agarwal P (2011). Prevalence of restless legs syndrome in North American and Werstern European populations: a systematic review. Sleep Medicine.

[8] Hening W, Walters AS, Allen RP, Montplaisir J, Myers A, Ferini-Strambi L (2004). Impact, diagnosis and treatment of restless legs syndrome (RLS) in a primary care population: the REST (RLS epidemiology, symptoms, and treatment) primary care study. Sleep Medicine.

[9] Collado-Seidel V, Winkelmann J, Trenkwalder C (1999). Aetiology and treatment of restless legs syndrome. CNS Drugs.

[10] Enomoto M, Inoue Y, Namba K, Munezawa T, Matsuura M (2008). Clinical characteristics of restless legs syndrome in end-stage renal failure and idiopathic RLS patients. Movement Disorders.

[11] Kutner NG, Zhang R, Huang Y, Bliwise DL (2012). Racial differences in restless legs symptoms and serum ferritin in an incident dialysis patient cohort. International Urology and Nephrology.

[12] DeFerio JJ, Govindarajulu U, Brar A, Cukor D, Lee KG, Salifu MO (2017). Association of restless legs syndrome and mortality in end-stage renal disease: an analysis of the United States Renal Data System (USRDS). BMC Nephrology.

[12] Ferreira KF, Eckeli A, Dach F, Schwalbach MT, Schwalbach J, Speciali JG (2013). Prevalence of restless legs syndrome in patients with chronic pain in Maputo, Mozambique. Sleep Medicine.

[13] Collado-Seidel V, Kohnen R, Samtleben W, Hillebrand GF, Oertel WH, Trenkwalder C (1998). Clinical and biochemical findings in uremic patients with and without restless legs syndrome. American Journal of Kidney Diseases.

[13] Burtscher C, Baxmann A, Kassubek J (2014). Prevalence of restless legs syndrome in an urban population of eastern Africa (Tanzania). Journal of Neurological Sciences.

[14] Trenkwalder C, Allen R, Högl B, Paulus W, Winkelmann J (2016). Restless legs syndrome associated with major diseases: a systematic review and new concept. Neurology.

[15] Dhawan V, Ali M, Chaudhuri KR (2006). Genetic aspects of restless legs syndrome. Postgraduate Medical Journal.

[16] Trenkwalder C, Seidel VC, Gasser T, Oertel WH (1996). Clinical symptoms and possible anticipation in a large kindred of familial restless legs syndrome. Movement Disorders.

[17] Schormair B, Plag J, Kaffe M (2011). MEIS1 and BTBD9: genetic association with restless leg syndrome in end stage renal disease. Journal of Medical Genetics.

[18] Thyagarajan D (2008). Restless legs syndrome. Australian Prescriber.

[19] Gonzalez-Latapi P, Malkani R (2019). Update on restless legs syndrome: from mechanisms to treatment. Current Neurology and Neuroscience Reports.

[20] Stowe RC (2019). Acute drug-induced symptoms of restless legs syndrome in an emergency department: what's in a name?. Journal of Clinical Sleep Medicine.

[21] Mao S, Shen H, Huang S, Zhang A (2014). Restless legs syndrome in dialysis patients: a meta-analysis. Sleep Medicine.

[22] Cirignotta F, Mondini S, Santoro A, Ferrari G, Gerardi R, Buzzi G (2002). Reliability of a questionnaire screening restless legs syndrome in patients on chronic dialysis. American Journal of Kidney Diseases.

[23] Mitchell U, Hilton S (2014). Change of International Restless Legs Syndrome Study Group Rating Scale subscales with treatment and placebo: a pilot study. Journal of Parkonsinism and Restless Legs Syndrome.

[24] South African Renal Society (SARS) (2015). Guideline for the optimal care of patients on chronic dialysis in South Africa.

[26] Malaki M, Mortazavi FS, Moazemi S, Shoaran M (2012). Insomnia and limb pain in hemodialysis patients: what is the share of restless leg syndrome?. Saudi Journal of Kidney Disease and Transplantation.

[27] De Menezes AF, De Santa Motta DRM, Oliveira de Carvalho F (2018). Restless legs syndrome in dialysis patients: does the dialysis modality influence its occurrence and severity?. International Journal of Nephrology.

[28] Brzuszek A, Hazara AM, Bhandari S (2022). The prevalence and potential aetiological factors associated with restless legs syndrome in patients with chronic kidney disease: a cross-sectional study. International Urology and Nephrology.

[29] Aritake-Okada S, Nakao T, Komada Y (2011). Prevalence and clinical characteristics of restless legs syndrome in chronic kidney disease patients. Sleep Medicine.

[30] Rohani M, Aghaei M, Jenabi A, Yazdanfar S, Mousavi D, Miri S (2015). Restless legs syndrome in hemodialysis patients in Iran. Neurological Sciences.

[31] Chou JA, Kalantar-Zadeh K (2017). Volume balance and intradialytic ultrafiltration rate in the hemodialysis patient. Current Heart Failure Reports.

[32] Arnold R, Issar T, Krishnan AV, Pussell BA (2016). Neurological complications in chronic kidney disease. JRSM Cardiovascular Diseases.

[33] Matar SG, El-Nahas ZS, Aladwan H, Hasanin M, Elsayed SM, Nourelden AZ (2022). restless leg syndrome in hemodialysis patients: a narrative review. Neurologist.

[34] Jabbari B, Vaziri ND (2018). The nature, consequences, and management of neurological disorders in chronic kidney disease. Hemodialysis International.

[35] Araujo SMH, De Bruin VM, Nepomuceno LA (2010). Restless legs syndrome in end-stage renal disease: clinical characteristics and associated comorbidities. Sleep Medicine.

[36] Gade K, Blaschke S, Rodenbeck A, Becker A, Anderson-Schmidt H, Cohrs S (2013). Uremic restless legs syndrome (RLS) and sleep quality in patients with end-stage renal disease on hemodialysis: potential role of homocysteine and parathyroid hormone. Kidney and Blood Pressure Research.

[37] Szentkiralyi A, Molnar MZ, Czira ME (2009). Association between restless legs syndrome and depression in patients with chronic kidney disease. Journal of Psychosomatic Research.

[38] Bliwise DL, Zhang RH, Kutner NG (2014). Medications associated with restless legs syndrome: a case-control study in the US Renal Data System (USRDS). Sleep Medicine.

[39] Olgun Yazar H, Yazar T, Özdemir S, Kasko Arici Y (2019). Serum C-reactive protein/albumin ratio and restless legs syndrome. Sleep Medicine.

[40] eHealthMe Colchicine and restless leg syndrome–a phase IV clinical study of FDA data.

[41] Kerr PG, Van Bakel C, Dawborn JK (1989). Assessment of the symptomatic benefit of cool dialysate. Nephron.

